# Co-cultivation of *Lactobacillus acidophilus* and *Bacillus subtilis* mediates the gut-muscle axis affecting pork quality and flavor

**DOI:** 10.1186/s40104-025-01229-2

**Published:** 2025-07-02

**Authors:** Zhixin Lin, Xinchen Zhou, Tingting Lu, Wendong An, Shenghao Chen, Suchen Li, Hui Miao, Xinyan Han

**Affiliations:** 1 Key Laboratory of Animal Nutrition and Feed Science in East China, Ministry of Agriculture, College of Animal Science, Zhejiang University, Hangzhou, 310058 China; 2 Yazhou Bay Science and Technology City, Hainan Institute, Zhejiang University, Sanya, 572025 China

**Keywords:** Gut-muscle axis, Metagenome, Pork flavor, Pork quality, Probiotic

## Abstract

**Background:**

Pork quality and flavor are critical determinants of consumer preference, yet the role of gut microbiota in shaping meat characteristics remains underexplored. In this study, we investigated how a probiotic consortium (FAM: *Lactobacillus acidophilus* and *Bacillus subtilis*) modulates the gut-muscle axis to enhance pork flavor.

**Results:**

In finishing pigs, FAM supplementation significantly increased flavor-associated nucleotides and umami-enhancing amino acids in *longissimus dorsi* muscle. Metagenomic analysis revealed FAM-driven enrichment of glycan-degrading *Prevotella* and short-chain fatty acid-producing *Phascolarctobacterium*, accompanied by reduced antibiotic resistance genes and virulence factors. Spearman correlation linked *Prevotella copri* abundance with elevated muscle amino acids, suggesting microbial-encoded CAZymes as key mediators.

**Conclusions:**

This study provides the first evidence that probiotic-induced gut microbiota remodeling enhances pork flavor through metabolic cross-talk along the gut-muscle axis. The findings suggest a novel strategy for improving pork quality via dietary interventions targeting gut microbiota.

**Supplementary Information:**

The online version contains supplementary material available at 10.1186/s40104-025-01229-2.

## Introduction

Pork, an important part of the diet of people in many regions of the world, is not only a rich source of essential amino acids but also packed with vital micronutrients like iron, zinc, selenium, and a suite of B vitamins—including B_1_, B_2_, B_6_, and B_12_ [1]. With the escalating concern of consumers regarding healthy diets and the concurrent improvement in economic standards, there has been an increasing demand for superior quality and enhanced flavor in pork. Therefore, the pursuit of efficacious and innocuous methodologies to augment the quality and flavor of pork has emerged as a pivotal area of research.

The animal gut is a complex ecosystem consisting of host cells, gut microbiota, and available nutrients [2]. Among them, the gut flora is a complex and stable microbial community, and the homeostasis of the intestinal environment has been shown to be closely related to the body's defense against the invasion of intestinal pathogens [3]. However, recent research has revealed a novel pathway, the gut-muscle axis, suggesting that gut microbiota and its metabolites could potentially exert an influence on muscle function and mass [4, 5]. In clinical studies, the intestinal flora is thought to potentially influence muscle mass and function via specific gut-derived mediators such as short-chain fatty acids (SCFAs) and thus participate in the onset and development of sarcopenia [6, 7]. This research has extended to livestock and poultry, where probiotics have been shown to regulate gut flora, thereby enhancing muscle development and tenderness in lambs [8], and increasing the levels of flavor compounds in duck meat, improving its taste [9]. These findings underscore the significant potential for improving pork quality and flavor by modulating the gut microbiota.

Probiotics are widely used as feed additives in livestock and poultry production due to their beneficial outcomes in improving gut health and maintaining well-being [10]. Various potential mechanisms of action of probiotics have been proposed, especially mediating the modulatory effects of intestinal flora on host health, which mainly include competitive inhibition of pathogens and stimulation of the host's adaptive immune system [11, 12]. Despite the growing interest, there is a noticeable gap in the literature regarding the impact of probiotics on meat quality and flavor. FAM, a complex containing *Bacillus subtilis* and *Lactobacillus acidophilus* and their fermentation products, was shown to modulate intestinal microbial composition and promote the production of total SCFAs and butyric acid in weaned piglets, thereby improving intestinal barrier and immune function [13]. This study aims to investigate the role of dietary FAM in pork flavor and intestinal flora, exploring the effect and potential mechanisms by which FAM could improve meat flavor through its influence on the gut microbiota. Such research holds promise for developing innovative strategies to enhance pork flavor, capitalizing on the interaction between the gut microbiome and meat quality.

## Materials and methods

### Experimental design, animals, and diets

A total of 120 male finishing pigs with similar initial body weights (average weight 100.0 ± 8.4 kg, Duroc × Landrace × Yorkshire cross, provided by Hainan Nongken Swine Co., Ltd., Hainan, China) were randomly assigned to two groups, with three replicates and twenty pigs each. The control group (CON) was fed a corn-soybean meal type basal diet, and the FAM group was fed a basal diet supplemented with 0.1% FAM^®^ (provided by Zhejiang Kangwandechuan Technology Co., Ltd., mainly containing *Lactobacillus acidophilus* (≥ 1 × 10^6^ CFU/g) and *Bacillus subtilis* (≥ 1 × 10^6^ CFU/g) and their fermentation products). The basal diet was formulated to meet the nutritional standards set by the National Research Council (NRC, 2012) [14] for finishing pigs, with details provided in Table S1. Pigs were fed twice daily at 7:00 and 14:00, with ad libitum access to feed and water, and the barn was maintained clean daily. The barn temperature was maintained at 22 ± 2 °C with 60%–70% humidity throughout the experimental period. The total experimental period was 49 d. The average final weight of the pigs at the end of the experiment was 145.4 ± 14.8 kg. The animal experiment was approved by the Animal Care and Use Committee of Zhejiang University (permit number: ZJU20230042) and all experimental procedures conformed to the institutional guidelines for animal study. All efforts were made to minimize suffering.

### Sample collection

At the end of the 49-day experimental period, six medium-weight pigs were selected from each treatment group, with two pigs from each replication (*n* = 6 per group). The pigs were humanely slaughtered following standard operating procedures. After electrocution and exsanguination, the pigs were disemboweled using a sterile scalpel, and the contents of the mid-colon were carefully collected and transferred into 1.5-mL freezing tubes for later analysis. Subsequently, the *longissimus dorsi* muscle was extracted from the left half of each carcass at the level of the 3^rd^ to 4^th^ thoracic vertebrae. Approximately 200 g of this muscle was sampled to determine the levels of amino acids, flavor-nucleotides, and long-chain fatty acids. All collected samples were immediately snap-frozen in liquid nitrogen and subsequently stored at −80 °C for further analysis.

### Analysis of meat quality

The pH values of the *longissimus dorsi* muscle were determined at 24 h post-mortem, using a portable pH meter (Mettler Toledo S2-Food Ki, Mettler Toledo, Columbus, OH, USA). Subsequently, the meat color parameters, L* (lightness), a* (redness), and b* (yellowness), were sequentially determined 24 h after slaughtering by a meat colorimeter (Konica Minolta Chroma Meter CR 400, Osaka, Japan). The drip loss was evaluated in accordance with the EZ-DripLoss method [15]. Firstly, the meat was cut to an approximate thickness of 8 cm. Subsequently, its peripheral muscle membrane was carefully removed and then the meat was trimmed along the muscle fiber direction to form a cube with dimensions of 2 cm × 2 cm × 2 cm. After that, the sample was weighed (marked as *m*_1_) using an analytical balance. The EZ-measurement tubes were numbered and placed into the test tube rack. Subsequently, the sample was placed into the tube and then stored at 4 °C for 24 h. Following this period, the tube was retrieved, the surface liquid of the sample was blotted dry with filter paper, and the sample was weighed again (marked as *m*_2_). Finally, the drip loss rate was calculated using the formula: Drip loss rate = (*m*_1_− *m*_2_)/*m*_1_ × 100%. The intramuscular fat (IMF) content of pork was determined according to the Soxhlet extraction method. Briefly, a 3-g pork sample was minced, dried at 105 °C until constant weight, and then placed into a filter paper tube. The processed sample was placed in a Soxhlet extractor and extracted with anhydrous ether; the solvent was recovered by distillation after the extraction was completed, and finally the extractor bottle was dried to constant weight. The IMF content of pork was calculated according to the formula (IMF content = fat weight/sample weight × 100%).

### Myofiber morphology and immunofluorescence analysis

Muscle samples were rinsed with saline. After dehydration and embedding, the sections were stained with eosin hematoxylin and imaged using Nikon Eclipse Ci-L (NIKON Corporation, Tokyo, Japan). With Image-Pro Plus 6.0 software, five muscle fiber diameters per section were measured, and the total myofiber area and number in each image were determined to calculate the area and density of individual myofibers. For immunofluorescence analysis, paraffin-embedded muscle sections were dewaxed and rehydrated to expose antigens. They were incubated overnight at 4 °C with the primary antibody. Specifically, for the fast myosin heavy chain (MHC), we used a mouse anti-fast MHC primary antibody (clone MY-32, Sigma-Aldrich, St. Louis, MO, USA; 1:200 dilution). For the slow MHC, we used a rabbit anti-slow MHC primary antibody (ab11083, Abcam, Cambridge, UK; 1:150 dilution). After washing with PBS to remove unbound antibodies, the secondary antibody (Alexa Fluor 488-conjugated goat anti-mouse IgG (A-11001, Invitrogen, Carlsbad, CA, USA; 1:200, and Alexa Fluor 594-conjugated goat anti-rabbit IgG (A-11012, Invitrogen; 1:200)) was added and incubated for 50 min at room temperature. Then the muscle fibers were stained with DAPI in the dark for 10 min and washed three times with PBS in the dark. Finally, the samples were observed under Nikon Eclipse Ci-L, and the numbers of fast and slow myofibers in three fields of view per section were counted, with their percentages calculated using Image-Pro Plus 6.0 software.

### Analysis of muscle flavor content

Muscle samples were vacuum-frozen to determine amino acid content and then sieved to ensure homogeneity. For the analysis, 0.5 g of the sieved sample was placed in an ice-water bath within a 0 °C refrigerator, and 5 mL of phenol peroxyformate reagent was added. The mixture was allowed to react for 16 h to facilitate the oxidation of the sample. Excess oxidizing reagent was quenched with sodium metabisulfite. Following this, 25 mL of hydrochloric acid-phenol solution was added to the sample, which was then hydrolyzed in an oven at 110 °C for 24 h. Upon completion of the hydrolysis, 15 mL of a leucine standard solution was accurately added to each sample. After thorough mixing, 125 mL of citrate buffer and 19 mL of aqueous sodium hydroxide were added to adjust the pH of the solution to 2.2. The samples were subsequently analyzed using a Biochrom Bio 31+ Amino Acid Analyzer (Biochrom Ltd., Cambridge, England) under the following conditions: a 65-min run at 135 °C with simultaneous detection at 570 nm and 440 nm.

Extraction and derivatization of fat from muscle samples were performed according to the protocol of Folch et al. [16] and Laurentius et al. [17]. Briefly, 1 g of sample was homogenized with 10 mL of methanol and a small amount of pickling sand for 1 min, then 20 mL of chloroform was added and homogenization continued for an additional 2 min. The mixture was then filtered, and the residue was treated with 30 mL of a chloroform–methanol mixture (2:1, v/v). The filtrate was washed with 20 mL of chloroform and 10 mL of methanol. To separate the phases, 1/4 volume of the filtrate was mixed with water, followed by the addition of a methanol–water mixture (1:1, v/v), which was approximately 1/4 the volume of the lower layer. This washing step was repeated twice. The organic phase was evaporated to dryness under reduced pressure to give a fatty acid solution. The extracted fatty acid solution was derivatized with 2-hydrazinopyrimidine (purchased from Tokyo Chemical Industry, Tokyo, Japan), centrifuged, and the supernatant was collected for analysis using helium carrier gas at 0.5 MPa partial pressure. The samples were analyzed using a 6890 N gas chromatograph (Agilent Technologies, Santa Clara, CA, USA) equipped with a flame ionization detector. The column temperature was programmed to increase from 145 °C to 240 °C at a rate of 3 °C/min, with a helium flow rate of 1.4 mL/min. The separated fatty acids were then introduced into a 5973 N quadrupole mass spectrometer (Agilent Technologies, Santa Clara, CA, USA) operating in selective ion monitoring (SIM) mode for quantitative analysis.

Nucleotides in muscle were extracted according to the established protocol [18]. Nucleosides were then separated by reversed-phase ultra-high performance liquid chromatography using a Nexera LC-40 system (Shimadzu Corporation, Kyoto, Japan) on a Synergi™ Fusion-RP C18 column (250 mm × 2 mm, 4 μm particle size, 80 Å). Detection was performed using a triple quadrupole 8060X (Shimadzu Corporation, Kyoto, Japan) in ESI source positive ion mode using multiple reaction monitoring (MRM) modes.

### Metagenome sequencing of colonic microbiota

A sample of 0.5 g of colon contents was taken, and genomic DNA was extracted using a DNA extraction kit (FastDNA^®^ Spin Kit for Soil, MP Biomedicals) according to the manufacturer’s instructions. The extracted genomic DNA was verified by 1% agarose gel electrophoresis. Sequencing libraries were prepared by fragmenting the DNA to approximately 350 bp using a Covaris M220 instrument. Paired-end (PE) libraries were constructed and subjected to bridge PCR amplification. Raw Illumina sequencing data underwent stringent quality control: low-quality reads (average Phred score < 20 over a sliding window of 50 bp), reads containing adapter sequences, and reads with > 5% ambiguous bases (N) were removed using Trimmomatic v0.39. Potential chimeric sequences were identified and filtered using UCHIME v8.1 within the VSEARCH pipeline. High-quality reads were assembled using MEGAHIT v1.2.9 with default parameters. The assembled contigs were quality-filtered and aligned against a non-redundant gene catalog using SOAPaligner (http://soap.genomics.org.cn/). The non-redundant gene sets were compared with the NR database (amino acid sequence database of non-redundant proteins) using DIAMOND software (http://ab.inf.uni-tuebingen.de/software/diamond/) with a BLASTP comparison type, allowing for species identification based on taxonomic information. Species abundance was calculated by summing the corresponding gene abundances and was quantified at each taxonomic level (Domain, Phylum, Species) to create an abundance profile. Gene sequences were then annotated using COG, KEGG, CAZymes, ARDB, and VFDB databases and analyzed for species and functional differences.

### Statistical analysis

Data were statistically analyzed using SPSS 26.0 (SPSS Institute, Chicago, USA). All data were expressed as mean ± standard deviation (SD), followed by one-way ANOVA for comparing the differences between groups and Duncan's multiple range test for analyzing multiple comparisons. *P* < 0.05 were considered statistically significant. Values within 0.05 ≤ *P* ≤ 0.10 were interpreted as indicative of a statistical tendency, reflecting potential biological relevance that may warrant further investigation despite not meeting strict significance thresholds.

## Results

### Pork quality

As shown in Table 1, the drip loss of pork in the FAM group was lower than that in the CON group (*P* < 0.05), whereas there were no significant changes in pH_24h_, meat color, and IMF content (*P* > 0.05).

**Table 1:** Meat quality of the pig’s *longissimus dorsi* muscle

Item	CON^1^	FAM^1^	*P*-value
pH_24h_	5.63 ± 0.08	5.64 ± 0.05	0.641
Drip loss, %	8.22 ± 0.33^a^	7.55 ± 0.29^b^	0.004
Intramuscular fat, %	1.26 ± 0.28	1.31 ± 0.05	0.729
Meat color			
L*	43.18 ± 1.08	43.35 ± 0.98	0.779
a*	4.45 ± 0.31	4.66 ± 0.23	0.212
b*	5.14 ± 0.28	5.27 ± 0.23	0.365

^1^CON group, basal diet; FAM group, basal diet added with 0.1% FAM

Values are mean ± SD, *n* = 6. Mean values within a row with different superscript letters denote statistical significantly difference (*P* < 0.05)

### Changes in the morphology and type of pork muscle fibers

**Fig. 1:**
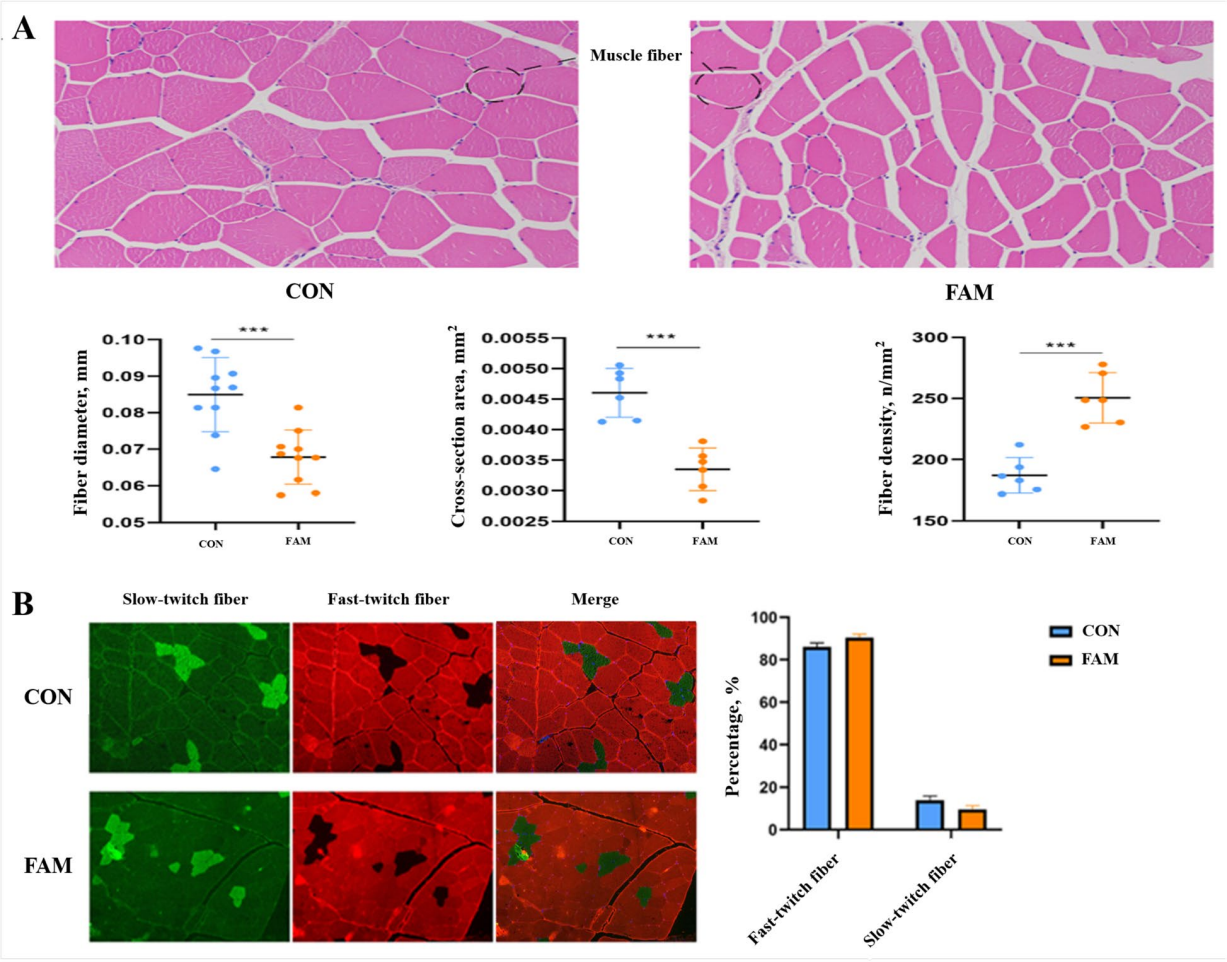
Muscle fiber morphology HE staining section (**A**); Immunofluorescence sections of muscle fibers (**B**). CON group, basal diet; FAM group, basal diet added with 0.1% FAM

Pork muscle fiber diameter and area were lower (*P* < 0.05) and density was higher (*P* < 0.05) in the FAM group compared to the CON group (Fig. 1A). As shown in Fig. 1B, there was no significant difference in the percentage of slow and fast twitch fibers by dietary addition of FAM compared to the control group (*P* > 0.05).

### Amino acid content in pork

The amino acid profiles of *longissimus dorsi* muscle demonstrated significant alterations in the FAM group compared to the CON group (Table 2). Fresh amino acids, including aspartate (Asp) and glutamate (Glu), exhibited marked increases in the FAM group. Similarly, sweet amino acids such as threonine (Thr), serine (Ser), and lysine (Lys) were elevated in FAM-fed pigs (*P* < 0.05). Among bitter amino acids, significant enhancements were observed for valine (Val), methionine (Met), isoleucine (Ile), leucine (Leu), arginine (Arg), tyrosine (Tyr), and histidine (His) in the FAM group (*P* < 0.05), while phenylalanine (Phe) showed only a marginal upward trend (*P* = 0.079).

**Table 2:** Content of amino acids in the pig’s *longissimus dorsi* muscle

Item, mg/g	CON^1^	FAM^1^	*P*-value
Fresh amino acids			
Aspartate (Asp)	1.53 ± 0.10^b^	1.72 ± 0.07^a^	0.003
Glutamate (Glu)	2.49 ± 0.13^b^	2.81 ± 0.15^a^	0.006
Sweet amino acids			
Threonine (Thr)	0.75 ± 0.06^b^	0.86 ± 0.05^a^	0.006
Serine (Ser)	0.65 ± 0.05^b^	0.76 ± 0.04^a^	0.005
Glycine (Gly)	0.81 ± 0.05	0.81 ± 0.06	0.905
Proline (Pro)	0.77 ± 0.04	0.79 ± 0.06	0.466
Alanine (Ala)	0.97 ± 0.08	1.05 ± 0.06	0.101
Lysine (Lys)	1.50 ± 0.09^b^	1.70 ± 0.10^a^	0.004
Bitter amino acids			
Valine (Val)	0.76 ± 0.07^b^	0.90 ± 0.06^a^	0.011
Methionine (Met)	0.44 ± 0.03^b^	0.50 ± 0.03^a^	0.009
Isoleucine (Ile)	0.75 ± 0.06^b^	0.86 ± 0.06^a^	0.010
Leucine (Leu)	1.30 ± 0.08^b^	1.51 ± 0.08^a^	0.003
Arginine (Arg)	1.00 ± 0.07^b^	1.19 ± 0.07^a^	0.003
Tyrosine (Tyr)	0.56 ± 0.04^b^	0.67 ± 0.05^a^	0.007
Phenylalanine (Phe)	0.68 ± 0.06	0.75 ± 0.04	0.079
Histidine (His)	0.67 ± 0.05^b^	0.72 ± 0.03^a^	0.031

^1^CON group, basal diet; FAM group, basal diet added with 0.1% FAM

Values are mean ± SD, *n* = 6. Mean values within a row with different superscript letters denote statistical significantly difference (*P* < 0.05)

### Flavor-presenting nucleotides in pork

**Fig. 2:**
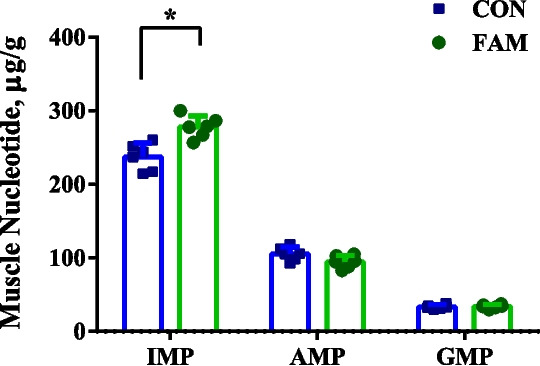
Content of flavor-presenting nucleotides in pig’s *longissimus dorsi* (*n* = 6). CON group, basal diet; FAM group, basal diet added with 0.1% FAM. AMP: adenosine monophosphate; GMP: guanosine 5'-monophosphate; IMP: inosine 5'-monophosphate

The content of IMP was higher in the FAM group than in the CON group (*P* < 0.05), and there was no difference in the content of AMP and GMP (*P* > 0.05) (Fig. 2).

### Long-chain fatty acids in pork

**Fig. 3:**
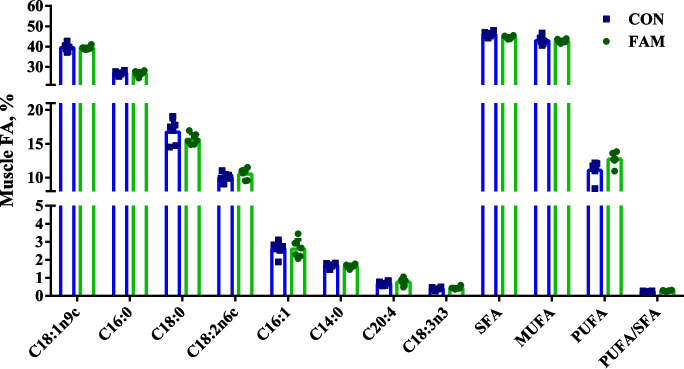
Content of long-chain fatty acids in pig’s *longissimus dorsi* (*n* = 6). CON group, basal diet; FAM group, basal diet added with 0.1% FAM

We examined a total of 37 fatty acids in the *longissimus dorsi*, of which 21 were detected. Among them, the eight most abundant fatty acids (including oleic acid, palmitic acid, stearic acid, linoleic acid, palmitoleic acid, myristic acid, arachidonic acid, and α-linolenic acid), as well as the total saturated fatty acids (SFAs), monounsaturated fatty acids (MUFAs), polyunsaturated fatty acids (PUFAs), and the PUFAs/SFAs ratio, are shown in Fig. 3. No significant differences were observed in the levels of long-chain fatty acids or the PUFAs/SFAs ratio between the FAM and CON groups (*P* > 0.05).

### Diversity and structure of intestinal flora

**Fig. 4:**
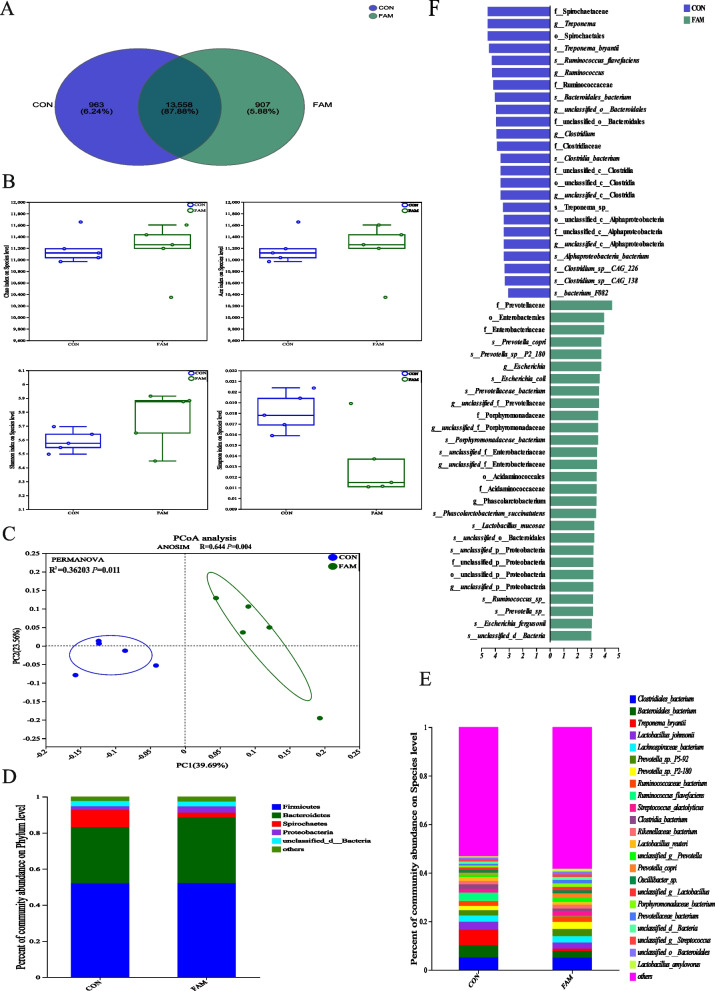
Diversity and structure of pig colonic microbiota. **A** Venn diagram of species numbers. **B** Alpha diversity index of the colonic microbiota. **C** Beta diversity analysis of the colonic microbiota. **D** Community structure of gut bacteria on Phylum level. **E** community structure of gut bacteria on Species-level. **F** LEfSe analysis. CON group, basal diet; FAM group, basal diet added with 0.1% FAM

To further investigate the changes in intestinal microorganisms in finishing pigs, metagenomic sequencing of colonic microbiota was performed. A total of 13,558 common microbial species were detected in the colon of pigs, with 963 unique species identified in the CON group and 907 in the FAM group (Fig. 4A). Analysis of α-diversity indices at the species level revealed no significant differences between the FAM and CON groups in Chao1, Ace, Shannon, or Simpson indices (*P* > 0.05), indicating comparable community richness and diversity (Fig. 4B). Principal Coordinates Analysis (PCoA) based on Bray-Curtis distances demonstrated distinct clustering of colonic microbiota between the two groups (Fig. 4C). ANOSIM analysis confirmed significant between-group differences (R = 0.644, *P* = 0.004), where R > 0 and *P* < 0.01 indicated that inter-group variation exceeded intra-group variation. Similarly, PERMANOVA analysis (R^2^ = 0.362, *P* = 0.011) supported the robustness of group separation, validating the structural divergence in microbial composition. At the phylum level, the main structures of the two groups were basically the same, with higher abundance of Firmicutes, Bacteroidota, Spirochaetes, and Proteobacteria, of which Firmicutes accounted for 52.15% in both groups and was the most predominant phylum in the intestinal bacterial group of finishing pigs (Fig. 4D). At the species level, *Clostridiales bacterium*, *Bacteroidales bacterium*, *Treponema bryantii*, *Lactobacillus johnsonii*, *Lachnospiraceae bacterium*, *Prevotella* sp. P5-92, *Prevotella* sp. P2-180, *Ruminococcaceae bacterium*, *Ruminococcus flavefaciens*, and *Streptococcus alactolyticus* were the top ten most abundant species. In the FAM group, *Bacteroidales bacterium*, *Treponema bryantii*, *Lactobacillus johnsonii*, *Ruminococcus flavefaciens* were less abundant than in the CON group. And the abundance of *Lachnospiraceae bacterium*, *Prevotella* sp. P5-92, *Prevotella* sp. P2-180, *Ruminococcaceae bacterium*, and *Streptococcus alactolyticus* in the FAM group were higher than that in the CON group (Fig. 4E).

LEfSe analysis showed that there were 29 marker species with higher abundance in the FAM group than the CON group from order to species, including *Prevotella* sp. P2-180, *Prevotella copri*, *Prevotellaceae bacterium*, *Porphyromonadaceae bacterium*, *Phascolarctobacterium succinatutens*, *Lactobacillus mucosae*, *Ruminococcus* (Fig. 4F).

### Functional analysis of the colonic microbiota

#### COG analysis

**Fig. 5:**
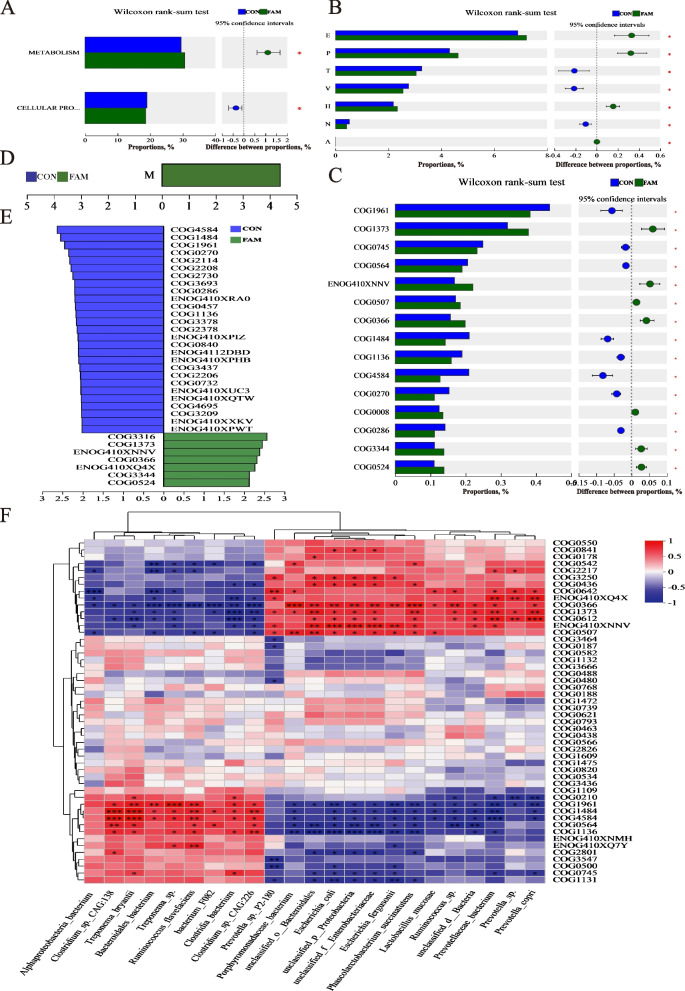
Analysis of COG function in colonic microbiota. **A–C** Wilcoxon rank-sum test from category to COG. **D** and **E** Linear discriminant analysis from function to COG, LDA > 2. **F** Correlation heatmap between gut bacterial signature species and COG function

The results of COG functional analysis of colonic Microbiota are shown in Fig. 5. Combined with Wilcoxon rank-sum test and LEfSe difference discriminant analysis, it can be seen that the metabolic functions of the intestinal flora in the FAM group were enhanced compared with those in the CON group (*P* < 0.05), including amino acid transport and metabolism (E), inorganic ion transport and metabolism (P), auxin transport and metabolism (H), RNA processing and modification (A), and cell wall/membrane/envelope biogenesis (M). The specificity of COG is demonstrated by the following: transposases (or inactivated derivatives) containing the DDE structural domain (COG3316), predicted ATPases of the AAA+ superfamily (COG1373), TonB-dependent receptor proteins involved in inorganic ion transport and metabolism (ENOG410XNNV), and deoxyribonuclease vα (COG0507), glycosidase/amylase (phosphorylase) family proteins (COG0366), glucosamine or glutamine tRNA synthetase (GlnS) involved in protein synthesis (COG0008), AAA-ATPase (ENOG410XQ4X), RNA-induced DNA polymerase (COG3344), and the ribokinase family of sugar- or nucleotide-kinases (COG0524).

Spearman correlation analysis of the enriched marker species with the top 50 functions in terms of total abundance at the COG level was performed, and the results are shown in Fig. 5F. The FAM group marker species *Porphyromonadaceae bacterium*, *Phascolarctobacterium succinatutens* were positively correlated with the abundance of glycosidases/amylases (phosphorylase) family (COG0366) abundance, *Prevotella* sp. P2-180 was positively correlated with histidine kinase (COG0642) abundance and negatively correlated with transposase (COG3547) abundance, *Prevotella copri* (COG0612) abundance was strongly positively correlated with peptidase (COG0612) abundance, *Prevotellaceae bacterium* was strongly negatively correlated with transposase (COG4584) abundance, positively correlated with AAA-ATPase (ENOG410XQ4X) abundance, and *Ruminococcus* was negatively correlated with pseudouridine synthase (COG0564) activity.

#### KEGG analysis

**Fig. 6:**
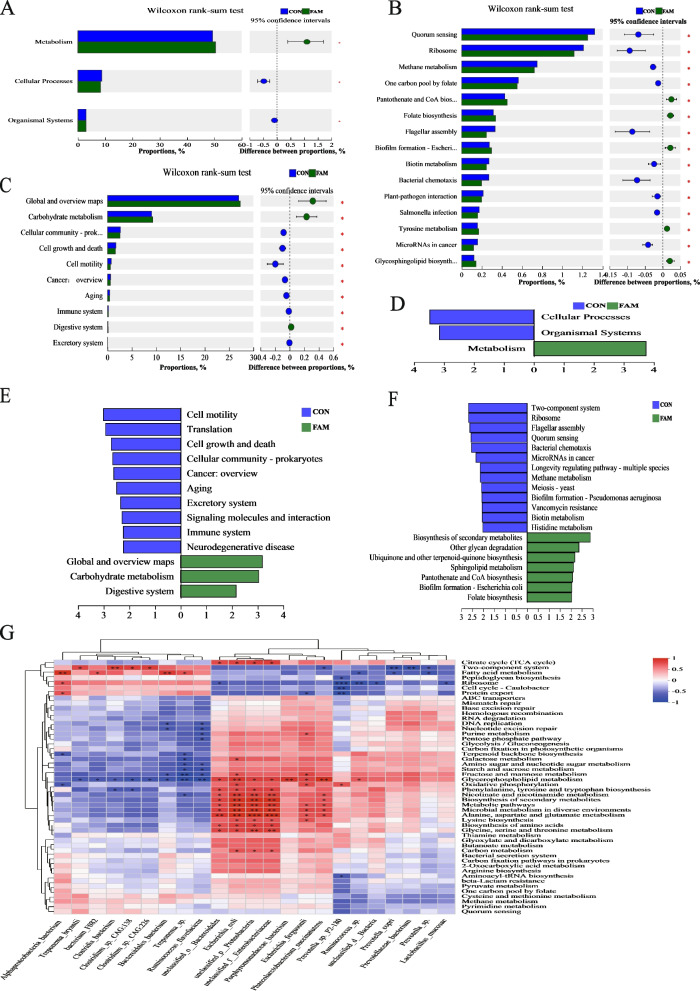
Analysis of KEGG function in colonic microbiota. **A–C** Wilcoxon rank-sum test of level 1–3. **D–F** Linear discriminant analysis of level 1–3, LDA > 2. **G** Correlation heatmap between gut bacterial signature species and KEGG function

The results of KEGG functional analysis of colonic microbiota are shown in Fig. 6. The combination of Wilcoxon rank-sum test and LEfSe difference discriminant analysis showed that the metabolic function was enhanced in the FAM group, in which the metabolic pathway overview and global view, glycan biosynthesis and metabolism, and digestive system functional pathways were enriched (*P* < 0.05), and 42 differential pathways were enriched at the Level 3, in which the biosynthesis of secondary metabolites, degradation of other glycans, and the pathways of pantothenic acid and coenzyme A metabolism, and differential analysis for amino acid metabolism showed that phenylalanine metabolism pathway was upregulated with alanine, aspartate and glutamate metabolism pathways and histidine metabolism pathway was downregulated with valine, leucine, and isoleucine degradation pathways in the FAM group.

Spearman correlation analysis of marker species with the top 50 total abundance of KEGG pathway Level 3 was performed, and the results are shown in Fig. 6G. The FAM group marker species *Porphyromonadaceae bacterium*, *Phascolarctobacterium succinatutens* positively correlated with glycerophospholipid metabolism, metabolic pathways, alanine, aspartate, and glutamate metabolism. *Prevotella* sp. P2-180 strongly negatively correlated with ribosomes, negatively correlated with Caulobacter cell cycle and protein export. *Prevotella copri*, *Prevotellaceae bacterium* negatively correlated with two-component system, and *Ruminococcus* was negatively correlated with ribosomes.

#### CAZymes analysis

**Fig. 7:**
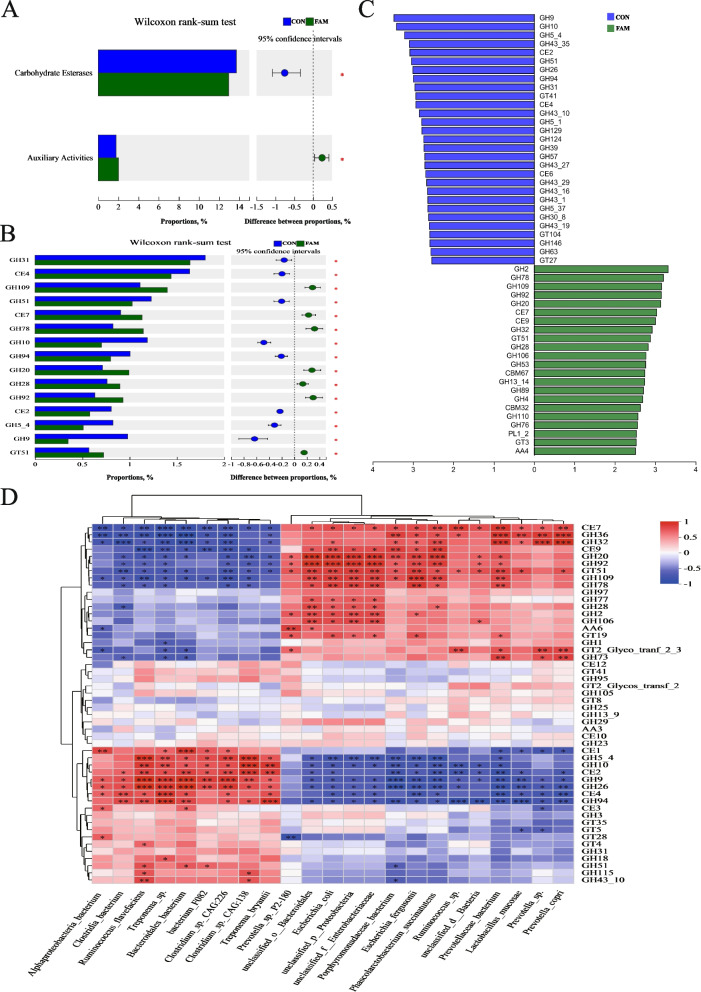
Analysis of CAZy function in colonic microbiota. **A** and **B** Wilcoxon rank-sum test from Class to Family. **C** Linear discriminant analysis of Family, LDA > 2.5. **D** Correlation heatmap between gut bacterial signature species and CAZy families

The results of the CAZy functional difference analysis of the intestinal bacterial group are shown in Fig. 7. Combined with the Wilcoxon rank-sum test and LEfSe difference discriminant analysis, it was shown that the FAM group was enriched in coactivating enzymes, and at LDA > 2.5, the mannanase family of enzymes catalyzing glycosidase hydrolysis of the corresponding sugar residues (GH2), the α-L-rhamnosidase family of enzymes (GH78), the exo-cleaving α-N-acetylgalactosaminidase family of enzymes (GH109), the α-mannosidase family of enzymes (GH92), β-N-acetylglucosaminidase family (GH20), carbohydrate esterase family 7 (CE7) and carbohydrate esterase family 9 (CE9), which are widely involved in biodegradation and biosynthesis processes, and glycosyltransferase family 51 (GT51), which is involved in a variety of biosynthesis pathways, were found to be increased in abundance (*P* < 0.05).

Spearman correlation analysis of marker species with the top 50 families in total CAZy abundance was performed, and the results are shown in Fig. 7D. The FAM group marker species *Porphyromonadaceae bacterium*, *Phascolarctobacterium succinatutens* were strongly positively correlated with the family of β-N-acetylglucosaminidases (GH20), carbohydrate esterase family 7 (CE7), carbohydrate esterase family 9 (CE9), and mannosidase family (GH92). *Prevotella* sp. P2-180 was positively correlated with 1,4-benzoquinone reductase family (AA6), and *Prevotella copri*, *Prevotellaceae bacterium* was strongly positively correlated with the α-galactosidase family (GH36) and the fructosyltransferase family (GH32), and *Ruminococcus* was strongly negatively correlated with the sugar phosphorylase family (GH94).

#### ARDB and VFDB analysis

**Fig. 8:**
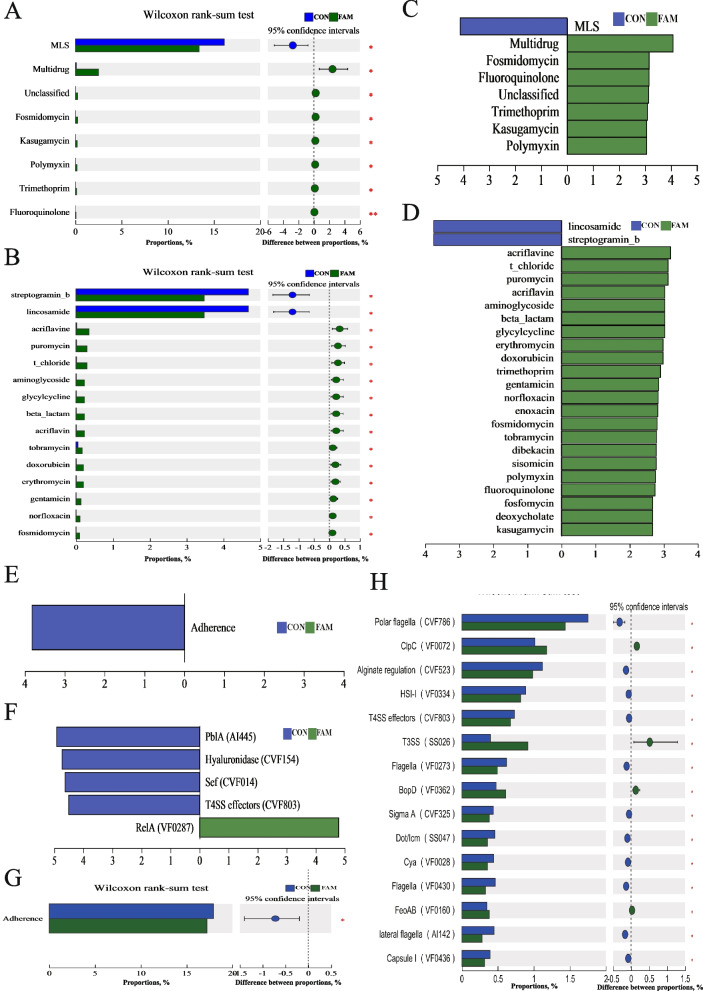
Analysis of ARDB and VFDB in colonic microbiota. **A** and **B** Wilcoxon rank-sum test from Antibiotic class to ARDB. **C** and **D** Linear discriminant analysis from Antibiotic class to Antibiotic type, LDA > 2. **E** and **F** Linear discriminant analysis from level 2 to VFs, LDA > 2; **G** and **H** Wilcoxon rank-sum test from level 2 to VFs

The results of the functional difference analysis of ARDB in the colonic microbiota are shown in Fig. 8A**–**D. The Wilcoxon rank sum test and LEfSe difference-in-differences discriminant analysis showed that FAM reduced the abundance of resistance genes against streptomycin and lincosamide antibiotics compared with the CON group (*P* < 0.05).

The results of the functional difference analysis of VFDB in the colonic microbiota are shown in Fig. 8E–H. Combined with the results of the Wilcoxon rank sum test and LDA discrimination, it can be seen that FAM reduced the abundance of virulence factors associated with adhesion compared with the CON group. For example, the abundance of PblA (AI445), hyaluronidase (CVF154), Sef (CVF014), Sef (CVF014), and T4SS effector (CVF803) were all decreased (*P* < 0. 05).

### Bacteriome and muscle amino acid correlation analysis

**Fig. 9:**
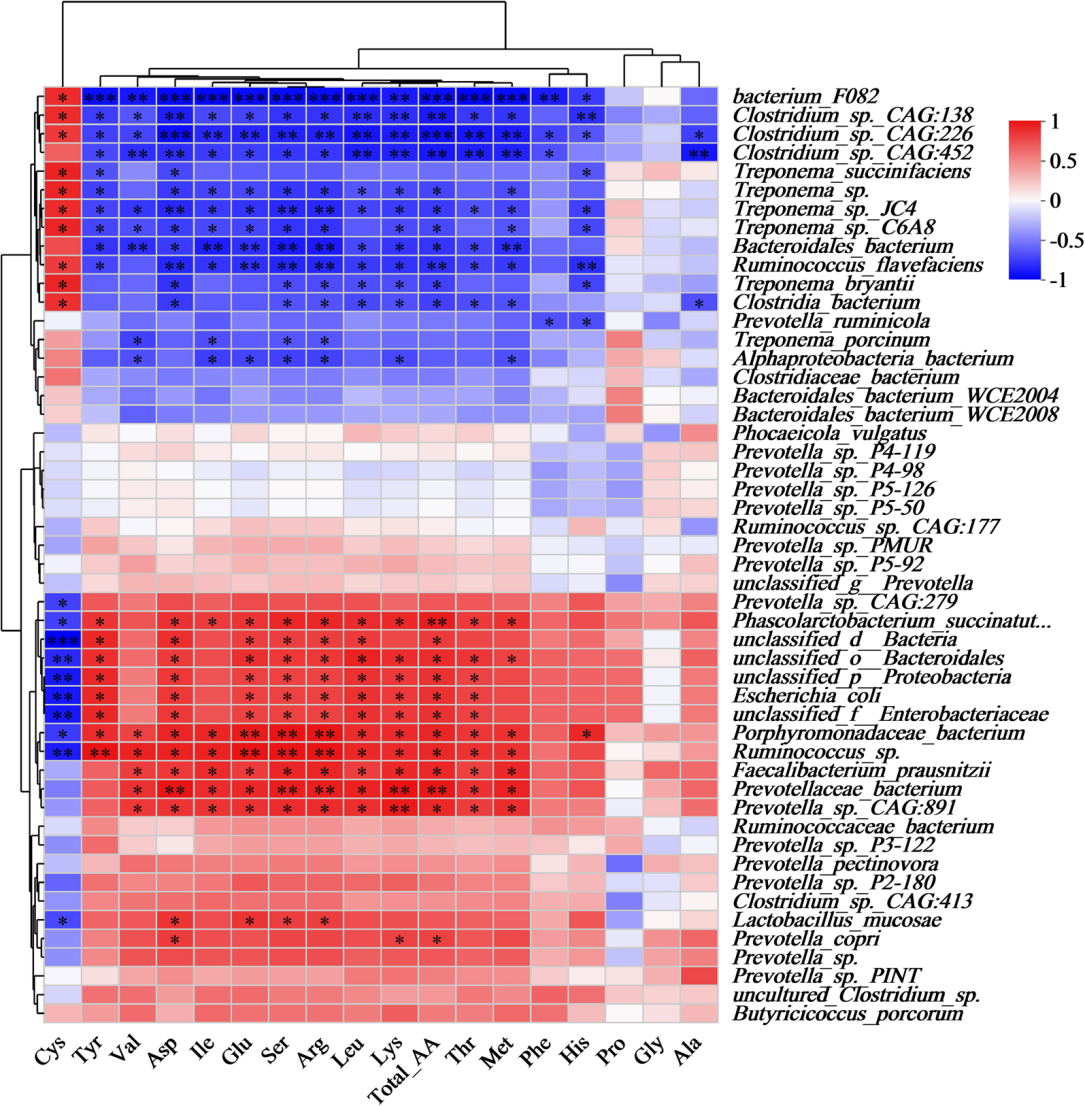
Correlation heatmap between colonic microbiota and muscle amino acid content

To analyze the correlation between meat flavor and colonic microorganisms, we used muscle amino acids (AA) as an environmental factor and subjected it to Spearman correlation analysis with the top 15 microbial species in terms of abundance (Fig. 9). Five species were significantly involved in amino acid metabolism, among which *Bacteroidales bacterium* was likely negatively correlated with muscle total AA, Asp, Lys, Glu, Met, Ile, Ser, and Arg content (*P* < 0.01), and possibly with muscle Val, Thr, and Leu content (*P* < 0.05); *Ruminococcus flavefaciens* was likely negatively correlated with muscle total AA, Asp, Glu, Ser, Arg, and His contents (*P* < 0.01), negatively correlated with muscle Tyr, Lys, Thr, Leu, Met, and Ile contents (*P* < 0.05), and positively correlated with muscle Cys contents (*P* < 0.05); *Treponema bryantii* may be negatively correlated with muscle total AA, Asp, Lys, Leu, Ser, Arg, and His content (*P* < 0.05) and positively correlated with muscle Cys content (*P* < 0.05); *Clostridia bacterium* may be negatively correlated with muscle total AA, Asp, Lys, Thr Leu, Met, Ser, Arg, and Ala content (*P* < 0.05), and positively correlated with muscle Cys content (*P* < 0.05); *Prevotella copri* may be positively correlated with muscle total AA, Asp, and Lys content (*P* < 0.05).

## Discussion

Our study establishes a novel linkage between probiotic-induced gut microbiota remodeling and pork quality, with drip loss reduction and umami-enhancing amino acid accumulation as key outcomes. These improvements align with the compact myofiber structure in FAM-fed pigs. Notably, while similar probiotic effects on meat texture were reported in lambs [8], the flavor-enhancing role of *Prevotella*-derived CAZymes appears unique to swine, possibly reflecting evolutionary divergence in host-microbe coadaptation to dietary fiber.

The quality attributes of pork, such as tenderness, intramuscular fat content, pH value, and drip loss, exert a direct and significant impact on the economic value of pork products. We found that FAM reduced the drip loss of the *longissimus dorsi*. It has been shown that water loss during post-mortem storage detrimentally affects the color of meat, accompanied by the concomitant loss of flavor substances and essential nutrients [19]. Consequently, drip loss serves as a crucial and reliable indicator of meat quality. In general, there exists a correlation between muscle fiber density and drip loss. A higher density of myofibers implies a more compact muscle structure, which restricts the seepage of water, thereby enabling the muscle to maintain enhanced water-holding capacity throughout the storage period [20]. Moreover, the type of muscle fiber also plays a role in determining drip loss. Typically, slow-twitch fibers possess a relatively high myoglobin content, are predominantly reliant on aerobic metabolism, exhibit a finer fiber diameter, and consequently possess a relatively strong water-holding capacity. In contrast, fast-twitch fibers are characterized by larger diameters, faster contraction rates, a predominantly anaerobic metabolic pathway, and a somewhat weaker water retention ability [21]. Correspondingly, in the present study, the myofiber diameter and area in the test pigs within the FAM group were found to be significantly lower compared to those in the CON group. Concomitantly, the myofiber density was observed to be elevated. However, no statistically significant alteration was detected in the proportion of fast and slow muscle fibers. These findings suggest that FAM is likely to enhance the water-holding capacity and reduce the drip loss of pork by increasing the density of myofibers, thereby contributing to an overall improvement in pork quality.

Flavor is a critical quality attribute of meat products, significantly impacting consumer acceptance and preference [22]. Amino acids, nucleotides, fatty acids, and other components in meat, known as flavor-active precursors, play a crucial role in flavor production [23]. Amino acids, in particular, serve as precursors in the Maillard reaction and Strecker degradation, contributing to the formation of volatile compounds that are key to meat's aroma when heated [24]. Inosine 5’-monophosphate (IMP), guanosine 5’-monophosphate (GMP), and adenosine monophosphate (AMP) are prominent among the flavor nucleotides. Fatty acids provide important fatty flavor to cooked meat [25, 26]. In the present study, we found that FAM significantly alters the levels of these amino acids and flavor-presenting nucleotides (especially IMP) in pork. IMP is a key flavor nucleotide that undergoes enzymatic degradation into inosine and hypoxanthine during post-mortem aging, both of which are critical contributors to umami taste and meat flavor intensity [27]. Its accumulation enhances the savory profile of pork by synergizing with glutamate and aspartate, amplifying the perception of sweetness and umami through the “flavor-enhancing effect” [28]. Furthermore, IMP’s degradation products participate in Maillard reaction pathways during cooking, generating heterocyclic compounds (e.g., pyrazines, thiazoles) that define roasted and grilled meat aromas [29].

Recently, several studies have proposed the existence of the gut-muscle axis to explain the interaction between the gut microbiota and muscle. The notion that the gut microbiota is strongly influenced by diet is well recognized [30]. Therefore, as a class of active microorganisms beneficial to the host, probiotics hold great promise as a potential strategy to improve meat quality. In previous studies, probiotics not only mediated gut microbes to promote muscle anabolism [31] but also improved lamb meat quality [8] and duck meat flavor [9]. Therefore, we analyzed the colonic bacterial groups of pigs in an attempt to reveal the correlation between gut microbial structure and function and pork flavor. The results showed that the structure of the gut microbiota of pigs did shift after FAM intake. The FAM group was enriched with *Prevotella copri*, *Porphyromonadaceae bacterium*, *Phascolarctobacterium succinatutens*, *Lactobacillus mucosae*, and *Ruminococcus*. Among them, *Prevotella copri* can interact with *Bifidobacterium infantis* and play a key role in glycan metabolism [32]. *Porphyromonadaceae bacterium* and *Ruminococcus* are recognized for their short-chain fatty acid production. *Phascolarctobacterium succinatutens* can be adapted to the intestinal environment by exclusively utilizing succinic acid produced by other bacterial species to produce propionic acid [33]. The core genome of *Lactobacillus mucosae* mainly encodes translation and transcription, amino acid biosynthesis, sugar metabolism, and defense functions [34]. Experiments in mice have also shown that *Lactobacillus mucosae* can lead to an increase in the levels of acetate, propionate, and butyrate [35]. Our findings suggest that FAM enriches the abundance of probiotics involved in critical metabolic pathways, such as short-chain fatty acid synthesis, thereby fostering a healthy intestinal microecology and potentially enhancing pork flavor.

Subsequently, we performed a predictive analysis of gut microbiome function to further explore gut-muscle axis-related metabolic pathways. We utilized the COG database, a repository of homologous protein annotations, to infer protein functions and identify specific metabolic pathways by aligning unknown sequences with known protein profiles. Additionally, we leveraged the KEGG database, which offers comprehensive insights into metabolic pathways, diseases, drugs, and biological system functions. Integrated multi-omics analysis demonstrated that FAM supplementation reprograms microbial metabolic networks, particularly enhancing glycan degradation (KEGG pathway ko00500) and amino acid biosynthesis (ko00250). The enrichment of *Prevotella copri*, a known utilizer of complex plant polysaccharides [32], correlated with upregulated CAZymes (GH20, GH92), which may liberate monosaccharides as substrates for cross-feeding mutualists like *Phascolarctobacterium*. This metabolic synergy likely elevates propionate (a key SCFA) [33], a modulator of hepatic gluconeogenesis that promotes muscle glycogen deposition [31], thereby improving post-mortem IMP retention.

Carbohydrates are pivotal in shaping the gut microbiota. While animals lack the necessary enzymes to break down most dietary carbohydrates, gut bacteria step in, degrading these complex carbohydrates and fermenting them into SCFAs. These SCFAs not only serve as an energy source for the host but also exhibit anti-inflammatory properties, modulate epigenetic remodeling, and influence host metabolism. The enzymes responsible for acting on carbohydrates, known as carbohydrate-active enzymes (CAZymes), target a wide range of substrates. The Carbohydrate Active Enzyme Database categorizes these enzymes into different protein families based on their amino acid sequence similarity in the structural domains. Our analysis revealed that the FAM group had higher levels of various carbohydrate enzymes, such as α-N-acetylgalactosaminidases, acetyl xyloglucan esterases, and α-L-rhamnosidase, compared to the control group. This indicates that FAM significantly enhances the degradation of carbohydrates by intestinal microorganisms, potentially improving the overall gut microbiota function.

The Antibiotic Resistance Genes Database (ARDB) catalogs genetic elements associated with bacterial antibiotic resistance. In this study, FAM reduced the abundance of resistance genes against streptomycin antibiotics and lincosamide antibiotics. Streptomycin, an effective antibiotic against Gram-positive bacteria, operates by inhibiting bacterial ribosomes [36]. Lincomycin A, featuring a unique eight-carbon sulfur sugar core methyl lincosamide and an N-methyl proline side chain, is widely used to combat *Actinobacillus pleuropneumoniae* (the causative agent of porcine pleuropneumonia) [37]. The reduction in these ARGs suggests a potential decline in the selective pressure for antibiotic resistance within the gut microbiome, possibly due to FAM’s competitive exclusion of pathogenic bacteria. The Virulence Factor Database (VFDB) documents genes encoding bacterial virulence factors. In our experiment, the FAM group exhibited a reduced abundance of several virulence factor genes. These include genes for Polar flagella, Flagella, and Lateral flagella, which are crucial for bacterial motility, environmental sensing, and localization [38, 39]. Additionally, we observed a downregulation in the abundance of the toxin RTX, linked to cytotoxicity [40], and cytotoxins Cya and HlyA, which can disrupt host cell function [41, 42]. Virulence factors related to bacterial metabolism or defense mechanisms, such as BapA, Capsule I, and Pyridine-2,6-dithiocarboxylic acid, were also less abundant. These changes imply that FAM may suppress pathogenicity by limiting microbial adherence and toxin production.

We also performed a Spearman correlation analysis between the 15 most abundant bacterial species and muscle amino acid content to explore the potential species most promising to influence amino acid metabolism. The results showed that *Prevotella copri*, the dominant bacterium in the FAM group, was positively correlated with total AA, Asp, and total Lys content in muscle. This correlation suggests that alterations in the gut bacterial community, particularly an increase in bacteria that promote amino acid synthesis, could enhance amino acid production within the gut microecology. Consequently, this increase may lead to higher amino acid deposition in the muscle through the gut-muscle axis, thereby potentially improving meat flavor.

## Conclusion

In conclusion, this study has shed some light on the strong correlation between gut microbiota and pork flavor, along with potential underlying mechanisms. FAM affects the composition and function of gut microbes in pigs, which in turn enhances pig health and metabolism, and ultimately modulates the gut-muscle axis to regulate pork flavor. Unlike prior studies focusing on poultry, our work provides the first evidence that probiotic-driven microbiota remodeling directly enhances pork flavor in swine, a species with distinct gut anatomy and microbial ecology. Specifically, while *Lactobacillus*-mediated SCFA production is conserved across livestock, the FAM-induced shift toward *Prevotella*-dominated glycan metabolism appears unique to pigs, potentially reflecting evolutionary adaptations to high-fiber diets. This divergence underscores the necessity of species-specific probiotic strategies. However, the limitation of the experiments is the inability to directly demonstrate the effect of gut microbiota on meat quality and flavor. Future studies should employ fecal microbiota transplantation (FMT) to swine ileal-loop models, coupled with metabolomic profiling of portal vein blood, to establish direct microbiota-muscle metabolite transfer.

## Supplementary Information

Supplementary Material 1. Table S1. Ingredient composition and nutrient levels of the basal diets (air-dry basis), %

## Data Availability

The datasets during the current study are available from the corresponding author on reasonable request.

## Abbreviations

AA: Amino acid
AMP: Adenosine monophosphate
ARDB: Antibiotic Resistance Genes Database
CAZymes: Carbohydrate-active enzymes
COG: Clusters of Orthologous Groups
GMP: Guanosine 5'-monophosphate
IMP: Inosine 5'-monophosphate
IMF: Intramuscular fat
KEGG: Kyoto Encyclopedia of Genes and Genomes
MUFAs: Monounsaturated fatty acids
PUFAs: Polyunsaturated fatty acids
SCFAs: Short-chain fatty acids
SFAs: Saturated fatty acids
VFDB: Virulence Factor Database

## References

[1] Pereira PM, Vicente AF. Meat nutritional composition and nutritive role in the human diet. Meat Sci. 2013;93(3):586–92. 23273468 10.1016/j.meatsci.2012.09.018

[2] Illiano P, Brambilla R, Parolini C. The mutual interplay of gut microbiota, diet and human disease. FEBS J. 2020;287(5):833–55. 31955527 10.1111/febs.15217

[3] Qiu P, Ishimoto T, Fu L, Zhang J, Zhang Z, Liu Y. The gut microbiota in inflammatory bowel disease. Front Cell Infect Microbiol. 2022;12:733992. 35273921 10.3389/fcimb.2022.733992 PMC8902753

[4] Mancin L, Wu GD, Paoli A. Gut microbiota-bile acid-skeletal muscle axis. Trends Microbiol. 2023;31(3):254–69. 36319506 10.1016/j.tim.2022.10.003

[5] Ticinesi A, Lauretani F, Milani C, Nouvenne A, Tana C, Del Rio D, et al. Aging gut microbiota at the cross-road between nutrition, physical frailty, and sarcopenia: is there a gut-muscle axis? Nutrients. 2017;9(12):1303. 10.3390/nu9121303 PMC5748753 29189738

[6] Liu C, Cheung WH, Li J, Chow SK, Yu J, Wong SH, et al. Understanding the gut microbiota and sarcopenia: a systematic review. J Cachexia Sarcopenia Muscle. 2021;12(6):1393–407. 34523250 10.1002/jcsm.12784 PMC8718038

[7] Yin Y, Guo Q, Zhou X, Duan Y, Yang Y, Gong S, et al. Role of brain-gut-muscle axis in human health and energy homeostasis. Front Nutr. 2022;9:947033. 36276808 10.3389/fnut.2022.947033 PMC9582522

[8] Dou L, Liu C, Chen X, Yang Z, Hu G, Zhang M, et al. Supplemental *Clostridium butyricum* modulates skeletal muscle development and meat quality by shaping the gut microbiota of lambs. Meat Sci. 2023;204:109235. 10.1016/j.meatsci.2023.109235 37301103

[9] Xu L, Mao T, Xia M, Wu W, Chen J, Jiang C, et al. New evidence for gut-muscle axis: Lactic acid bacteria-induced gut microbiota regulates duck meat flavor. Food Chem. 2024;450:139354. 38636385 10.1016/j.foodchem.2024.139354

[10] Kober A, Riaz Rajoka MS, Mehwish HM, Villena J, Kitazawa H. Immunomodulation potential of probiotics: a novel strategy for improving livestock health, immunity, and productivity. Microorganisms. 2022;10(2):388. 10.3390/microorganisms10020388 PMC8878146 35208843

[11] Piewngam P, Zheng Y, Nguyen TH, Dickey SW, Joo HS, Villaruz AE, et al. Pathogen elimination by probiotic *Bacillus* via signalling interference. Nature. 2018;562(7728):532–7. 10.1038/s41586-018-0616-y PMC6202238 30305736

[12] Maldonado Galdeano C, Cazorla SI, Lemme Dumit JM, Vélez E, Perdigón G. Beneficial effects of probiotic consumption on the immune system. Ann Nutr Metab. 2019;74(2):115–24. 30673668 10.1159/000496426

[13] Xie Z, Li M, Qian M, Yang Z, Han X. Co-Cultures of* Lactobacillus acidophilus* and *Bacillus subtilis* enhance mucosal barrier by modulating gut microbiota-derived short-chain fatty acids. Nutrients. 2022;14(21):4475. 10.3390/nu14214475 PMC9657225 36364738

[14] National Research Council. Nutrient Requirements of Swine: Eleventh Revised Edition. Washington, DC: The National Academies Press; 2012. p. 420.

[15] Kaić A, Janječić Z, Golub K, Potočnik K. Comparison between standardized and modified EZ-DripLoss determination methods in chicken breast meat. Animals (Basel). 2023;13(6):1054. 10.3390/ani13061054 PMC10044003 36978595

[16] Folch J, Lees M, Sloane Stanley GH. A simple method for the isolation and purification of total lipides from animal tissues. J Biol Chem. 1957;226(1):497–509. 13428781

[17] Laurentius T, Kob R, Fellner C, Nourbakhsh M, Bertsch T, Sieber CC, et al. Long-chain fatty acids and inflammatory markers coaccumulate in the skeletal muscle of sarcopenic old rats. Dis Markers. 2019;2019:9140789. 31354893 10.1155/2019/9140789 PMC6636585

[18] Elani HW, Starr JR, Da Silva JD, Gallucci GO. Trends in dental implant use in the U.S., 1999–2016, and projections to 2026. J Dent Res. 2018;97(13):1424–30. 30075090 10.1177/0022034518792567 PMC6854267

[19] Huff-Lonergan E, Lonergan SM. Mechanisms of water-holding capacity of meat: the role of postmortem biochemical and structural changes. Meat Sci. 2005;71(1):194–204. 22064064 10.1016/j.meatsci.2005.04.022

[20] Koomkrong N, Gongruttananun N, Boonkaewwan C, Noosud J, Theerawatanasirikul S, Kayan A. Fiber characteristics of pork muscle exhibiting different levels of drip loss. Anim Sci J. 2017;88(12):2044–9. 28730693 10.1111/asj.12859

[21] Klont RE, Brocks L, Eikelenboom G. Muscle fibre type and meat quality. Meat Sci. 1998;49:S219–29. 22060713

[22] de Araújo PD, Araújo WMC, Patarata L, Fraqueza MJ. Understanding the main factors that influence consumer quality perception and attitude towards meat and processed meat products. Meat Sci. 2022;193:108952. 36049392 10.1016/j.meatsci.2022.108952

[23] Ramalingam V, Song Z, Hwang I. The potential role of secondary metabolites in modulating the flavor and taste of the meat. Food Res Int. 2019;122:174–82. 31229070 10.1016/j.foodres.2019.04.007

[24] Sun A, Wu W, Soladoye OP, Aluko RE, Bak KH, Fu Y, et al. Maillard reaction of food-derived peptides as a potential route to generate meat flavor compounds: a review. Food Res Int. 2022;151:110823. 34980374 10.1016/j.foodres.2021.110823

[25] Bleicher J, Ebner EE, Bak KH. Formation and analysis of volatile and odor compounds in meat—a review. Molecules. 2022;27(19):6703. 10.3390/molecules27196703 PMC9572956 36235239

[26] Bravo-Lamas L, Barron LJR, Farmer L, Aldai N. Fatty acid composition of intramuscular fat and odour-active compounds of lamb commercialized in northern Spain. Meat Sci. 2018;139:231–8. 29459300 10.1016/j.meatsci.2018.02.006

[27] Mottram DS. Flavour formation in meat and meat products: a review. Food Chem. 1998;62(4):415–24.

[28] Zhang F, Klebansky B, Fine RM, Xu H, Pronin A, Liu H, et al. Molecular mechanism for the umami taste synergism. Proc Natl Acad Sci USA. 2008;105(52):20930–4. 19104071 10.1073/pnas.0810174106 PMC2606899

[29] Fuke S, Konosu S. Taste - active components in some foods: a review of Japanese research. Physiol Behav. 1991;49(5):863–8. 1679558 10.1016/0031-9384(91)90195-t

[30] Zmora N, Suez J, Elinav E. You are what you eat: diet, health and the gut microbiota. Nat Rev Gastroenterol Hepatol. 2019;16(1):35–56. 30262901 10.1038/s41575-018-0061-2

[31] Lahiri S, Kim H, Garcia-Perez I, Reza MM, Martin KA, Kundu P, et al. The gut microbiota influences skeletal muscle mass and function in mice. Sci Transl Med. 2019;11(502):eaan5662. 10.1126/scitranslmed.aan5662 PMC7501733 31341063

[32] Chang HW, Lee EM, Wang Y, Zhou C, Pruss KM, Henrissat S, et al. *Prevotella copri* and microbiota members mediate the beneficial effects of a therapeutic food for malnutrition. Nat Microbiol. 2024;9(4):922–37. 10.1038/s41564-024-01628-7 PMC10994852 38503977

[33] Watanabe Y, Nagai F, Morotomi M. Characterization of *Phascolarctobacterium succinatutens* sp. nov., an asaccharolytic, succinate-utilizing bacterium isolated from human feces. Appl Environ Microbiol. 2012;78(2):511–8. 10.1128/AEM.06035-11 PMC3255759 22081579

[34] Jia Y, Yang B, Ross P, Stanton C, Zhang H, Zhao J, et al. Comparative genomics analysis of *Lactobacillus mucosae* from different niches. Genes. 2020;11(1):95. 10.3390/genes11010095 PMC7016874 31947593

[35] Wang Q, Fang Z, Li L, Wang H, Zhu J, Zhang P, et al. *Lactobacillus mucosae* exerted different antiviral effects on respiratory syncytial virus infection in mice. Front Microbiol. 2022;13:1001313. 10.3389/fmicb.2022.1001313 PMC9459143 36090099

[36] Li Q, Pellegrino J, Lee DJ, Tran AA, Chaires HA, Wang R, et al. Synthetic group A streptogramin antibiotics that overcome Vat resistance. Nature. 2020;586(7827):145–50. 32968273 10.1038/s41586-020-2761-3 PMC7546582

[37] Zhang L, Kang Z, Yao L, Gu X, Huang Z, Cai Q, et al. Pharmacokinetic/pharmacodynamic integration to evaluate the changes in susceptibility of *Actinobacillus pleuropneumoniae* after repeated administration of danofloxacin. Front Microbiol. 2018;9:2445. 10.3389/fmicb.2018.02445 PMC6194310 30369920

[38] Kühn MJ, Edelmann DB, Thormann KM. Polar flagellar wrapping and lateral flagella jointly contribute to *Shewanella putrefaciens* environmental spreading. Environ Microbiol. 2022;24(12):5911–23. 10.1111/1462-2920.16107 35722744

[39] Merino S, Shaw JG, Tomás JM. Bacterial lateral flagella: an inducible flagella system. FEMS Microbiol Lett. 2006;263(2):127–35. 16978346 10.1111/j.1574-6968.2006.00403.x

[40] Sotomayor-Pérez AC, Ladant D, Chenal A. Disorder-to-order transition in the CyaA toxin RTX domain: implications for toxin secretion. Toxins. 2014;7(1):1–20. 25559101 10.3390/toxins7010001 PMC4303809

[41] Bendtzen K, Petersen J. Effect of cyclosporin A (CyA) on the immune response. CyA competitively inhibits the function of monocyte/macrophage-derived T-lymphocyte-activating factor(s). Immunol Lett. 1982;5(6):331–6. 6762338 10.1016/0165-2478(82)90123-7

[42] Schulz E, Schumann M, Schneemann M, Dony V, Fromm A, Nagel O, et al. *Escherichia coli* alpha-hemolysin HlyA induces host cell polarity changes, epithelial barrier dysfunction and cell detachment in human colon carcinoma Caco-2 cell model via PTEN-dependent dysregulation of cell junctions. Toxins. 2021;13(8):520. 10.3390/toxins13080520 PMC8402498 34437391

