# Supplementary material for: Co-cultivation of Lactobacillus acidophilus and Bacillus subtilis mediates the gut-muscle axis affecting pork quality and flavor
Source: J Anim Sci Biotechnol. 2025 Jul 2;16:93. doi: 10.1186/s40104-025-01229-2 (PMC12220238; doi:10.1186/s40104-025-01229-2)
Supplement: Supplementary file 1 — Supplementary Material 1. Table S1. Ingredient composition and nutrient levels of the basal diets (air-dry basis), % [file 40104_2025_1229_MOESM1_ESM.docx]

**Table S1** Ingredient composition and nutrient levels of the basal diets (air-dry basis), %

| **Items** | **Content** |
| --- | --- |
| Ingredients |  |
| Corn | 39.7 |
| Soybean meal | 14.4 |
| Wheat bran | 8.00 |
| Rice bran | 10.00 |
| Brown rice | 24.00 |
| L-Lys | 0.20 |
| L-Met | 0.25 |
| L-Thr | 0.46 |
| L-Trp | 0.17 |
| NaCl | 0.30 |
| Premix^1^ | 1.00 |
| Total | 100 |
| Nutrient levels^2^ |  |
| Digestive energy, MJ/kg | 12.58 |
| Crude protein | 15.33 |
| Crude fat | 2.67 |
| Non-phytate phosphorus | 0.72 |
| Lysine | 0.64 |
| Methionine | 0.22 |
| Threonine | 0.52 |
| Valine | 0.77 |

^1^ Premix provided per kilogram diet: VA 10,000 IU, VD_3_ 2,000 IU, VE 20 IU, VK 4 mg, niacin 20 mg, pantothenic acid 25 mg, VB_1_ 2 mg, VB_2_ 4 mg, VB_6_ 2 mg, VB_12_ 30 μg, biotin 0.5 mg, folic acid 0.9 mg, iron 80 mg, copper 7 mg, manganese 4 mg, zinc 100 mg, iodine 0.28 mg, selenium 0.30 mg

^2^ The nutrient levels are measured values, except for digestive energy, which is a calculated value
